# The orphan solute carrier SLC10A7 is a novel negative regulator of intracellular calcium signaling

**DOI:** 10.1038/s41598-020-64006-3

**Published:** 2020-04-29

**Authors:** Emre Karakus, Marie Wannowius, Simon Franz Müller, Silke Leiting, Regina Leidolf, Saskia Noppes, Stefan Oswald, Martin Diener, Joachim Geyer

**Affiliations:** 1 Institute of Pharmacology and Toxicology, Faculty of Veterinary Medicine, Justus Liebig University Giessen, 35392 Giessen, Germany; 2 Institute of Pharmacology, University of Greifswald, 17487 Greifswald, Germany; 3 Institute of Veterinary Physiology and Biochemistry, Faculty of Veterinary Medicine, Justus Liebig University Giessen, 35392 Giessen, Germany

**Keywords:** Physiology, Pathogenesis

## Abstract

SLC10A7 represents an orphan member of the Solute Carrier Family SLC10. Recently, mutations in the human *SLC10A7* gene were associated with skeletal dysplasia, amelogenesis imperfecta, and decreased bone mineral density. However, the exact molecular function of SLC10A7 and the mechanisms underlying these pathologies are still unknown. For this reason, the role of SLC10A7 on intracellular calcium signaling was investigated. SLC10A7 protein expression was negatively correlated with store-operated calcium entry (SOCE) via the plasma membrane. Whereas SLC10A7 knockout HAP1 cells showed significantly increased calcium influx after thapsigargin, ionomycin and ATP/carbachol treatment, SLC10A7 overexpression reduced this calcium influx. Intracellular Ca^2+^ levels were higher in the SLC10A7 knockout cells and lower in the SLC10A7-overexpressing cells. The SLC10A7 protein co-localized with STIM1, Orai1, and SERCA2. Most of the previously described human SLC10A7 mutations had no effect on the calcium influx and thus were confirmed to be functionally inactive. In the present study, SLC10A7 was established as a novel negative regulator of intracellular calcium signaling that most likely acts via STIM1, Orai1 and/or SERCA2 inhibition. Based on this, SLC10A7 is suggested to be named as negative regulator of intracellular calcium signaling (in short: RCAS).

## Introduction

Ca^2+^ is one of the most versatile second messengers in eukaryotic cells. It is involved in many cellular processes such as muscle contraction, vesicle exocytosis, cell proliferation and growth, and gene expression^1^. Whereas calcium influx into excitable cells is mainly mediated by voltage-gated calcium channels, the major entry pathway of calcium into non-excitable cells involves calcium release-activated calcium channels in the plasma membrane that allow store-operated calcium entry (SOCE)^2,3^.

Orail and the canonical transient receptor potential protein (TRPC) are two well-recognized store-operated Ca^2+^ channels^4^. While SOCE through Orail is dependent on activation and translocation of the stromal interaction molecule^1^ (STIM1), SOCE through TRPC can function in a STIM1-dependent or -independent manner^4^. These store-operated calcium channels are opened in response to calcium depletion of the endoplasmic reticulum (ER). Release of Ca^2+^ from the intracellular stores leads to activation of the ER Ca^2+^ sensor STIM1, which then interacts with Orai1 subunits to form a STIM-Orai complex that supports SOCE at a typically high selectivity for Ca^2+^ ^5–7^. A local increase in Ca^2+^ levels or gradual refilling of the calcium stores then inactivates the channels by negative feedback regulation^8,9^.

The sarcoplasmic/endoplasmic reticulum calcium ATPase (SERCA) transports Ca^2+^ from the cytosol into the SR/ER lumen to maintain the cytosolic Ca^2+^ at its low resting level^10^. The activity of SERCA is regulated according to the cellular requirements and extracellular signals of two different small proteins, phospholamban and sarcolipin. Both regulator proteins are only expressed in muscle cells, and decrease the calcium affinity of SERCA^11,12^. STIM/Orai activation, inactivation, and interaction are also regulated by several cellular factors. These include the CRAC channel regulator 2A (CRACR2A) that stabilises the STIM-Orai complex^13,14^, and the SOCE-associated regulatory factor (SARAF) that slowly inactivates STIM-dependent SOCE to prevent Ca^2+^ overfilling of the cell^15^.

In the present study, we identified an additional and novel negative regulator of intracellular calcium signaling, the orphan solute carrier SLC10A7, that most likely acts via STIM1, Orai1 and/or SERCA2 inhibition. Very recently, several mutations within the human *SLC10A7* gene were identified in patients with skeletal dysplasia, amelogenesis imperfecta and decreased bone mineral density^16–18^. These included the splice-site mutations c.774−1G > A (leading to skipping of exons 9 + 10 or only of exon 10), as well as c.773+1G > A and c.722-16A > G (both leading to skipping of exon 9), as well as the missense mutations c.388G > A (G130R), c.221T > C (L74P), c.335G > A (G112D) and c.908C > T (P303L)^16–18^. This pathological human phenotype was verified in (I) *Slc10a7*^*−/−*^ knockout mice, which show tooth enamel anomalies, shortened long bones, and growth plate disorganization^17^ and (II) in *Slc10a7*-deficient zebrafish, which show decreased calcium deposits in bone mineralization^16^. As patients with *SLC10A7* mutations revealed unique glycomic signatures and mis-localization of glycoproteins, a role of SLC10A7 in glyosaminoglycan synthesis, transport of glycoproteins to the extracellular matrix, and bone mineralization was suggested in these reports^16–18^. However, the exact molecular function of the SLC10A7 protein is still unclear, and the identified genomic *SLC10A7* mutations have not been analyzed and verified at the functional protein level so far. Therefore, in the present study, we have established SLC10A7 knockout and SLC10A7 overexpressing cell lines and show, for the first time, that SLC10A7 protein expression is negatively correlated with SOCE. Based on this, SLC10A7 is suggested to be named as negative regulator of intracellular calcium signaling (in short: RCAS).

## Results

### SLC10A7 knockout and overexpressing cell lines

**Figure 1:**
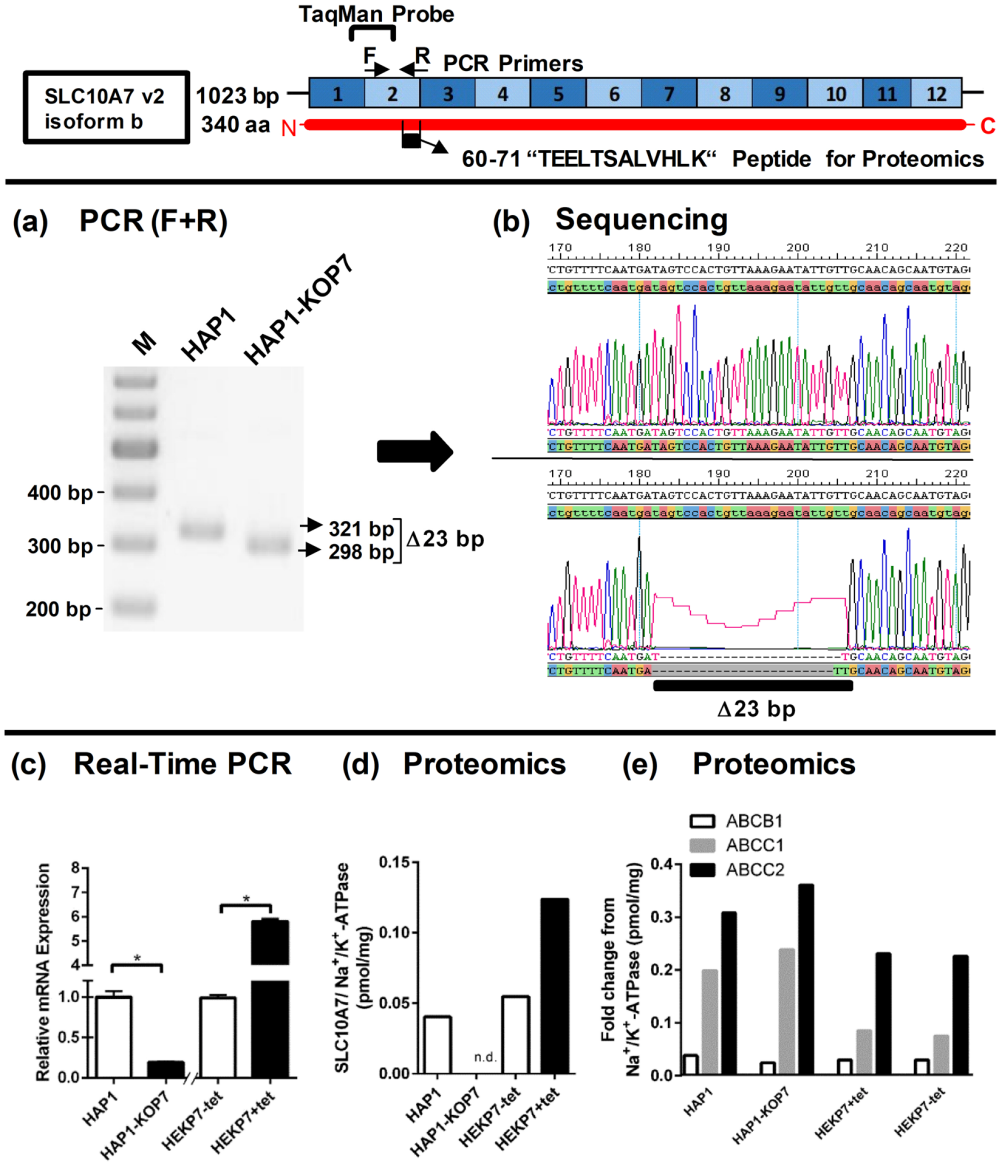
SLC10A7 mRNA and protein expression in the HAP1 and HEK293 cell lines. (**a**) Genomic DNA of HAP1 and HAP1-KOP7 cells was used for PCR amplification of the region flanking the site of CRISPR/Cas mutation in the *SLC10A7* coding exon 2 by using the oligonucleotide primers F and R. (**b**) PCR products of 321 bp (HAP1) and 298 bp (HAP1-KOP7) were subjected to DNA sequencing. The chromatograms clearly confirmed the CRISPR/Cas mutation of 23 bp within the reading frame of SLC10A7 in exon 2. (**c**) SLC10A7 mRNA expression was quantified by real-time PCR in the HAP1 and HEK293 cell lines using an SLC10A7-specific TaqMan probe targeting exon boundary 1→2. SLC10A7 expression was significantly lower in the HAP1-KOP7 cells compared to the HAP1 cells, but was significantly increased in the HEKP7 cells by tetracycline treatment (+tet). Data represent means ± RQmin/RQmax of n = 3 values (*p* < 0.05, Student’s t-test). (**d-e**) Protein abundance of the SLC10A7 (**d**) and ABCB1, ABCC1/2 (**e**) proteins was analyzed by mass spectrometry-based targeted proteomics.

In order to investigate the role of SLC10A7 for the influx of calcium into eukaryotic cells, we established cell models for SLC10A7 knockout as well as SLC10A7 overexpression, respectively. For the first approach, we used the near-haploid human cell line HAP1, which is derived from chronic myelogenous leukemia cells. Wild type HAP1 cells (HAP1), as well as CRISPR/Cas9-mediated SLC10A7 knockout HAP1 cells (HAP1-KOP7) were used. These HAP1-KOP7 cells revealed a genomic 23 bp deletion in coding exon 2, as shown by PCR amplification of the region of interest followed by DNA sequencing (Fig. 1a,b). Apart from destroying the coding sequence of the SLC10A7 protein, this mutation additionally seemed to compromise the stability of the *SLC10A7* transcript. Consequently, significantly lower *SLC10A7* mRNA expression levels in the HAP1-KOP7 cells were detected compared to the HAP1 wild type cells by means of real-time PCR expression analysis (Fig. 1c). Finally, the absence of SLC10A7 was confirmed on the protein level by mass spectrometry (MS)-based proteomics using the SLC10A7-specific reference peptide TEELTSALVHLK. In the HAP1-KOP7, this peptide could not be detected, but showed considerable presence in the HAP1 wild type cells (Fig. 1d). The second approach aimed to overexpress the SLC10A7 protein in cell culture. For this purpose, human embryonic kidney HEK293 cells, stably transfected with an SLC10A7 construct via Flp-FRT recombination, were used. Within these stably SLC10A7-transfected HEK293 cells (here referred to as HEKP7), SLC10A7 expression is under the control of a tetracycline-regulated promoter. Tetracycline treatment of these cells (HEKP7+tet) increased the *SLC10A7* mRNA expression several fold compared with non-tetracycline treated cells (HEKP7-tet). This was shown at the mRNA expression level via real-time PCR (Fig. 1c), and on the protein level by means of MS-based proteomics (Fig. 1d). As control groups, other membrane carriers (ABCB1, ABCC1, and ABCC2) were included in this analysis, and showed comparable expression levels in HAP1 wild type and HAP1-KOP7 cells, as well as in HEKP7+tet and HEKP7-tet, respectively (Fig. 1e). Interestingly, SLC10A7 protein expression was comparable between the HAP1 and HEKP7-tet cells (Fig. 1d).

### SLC10A7 regulates SOCE

**Figure 2:**
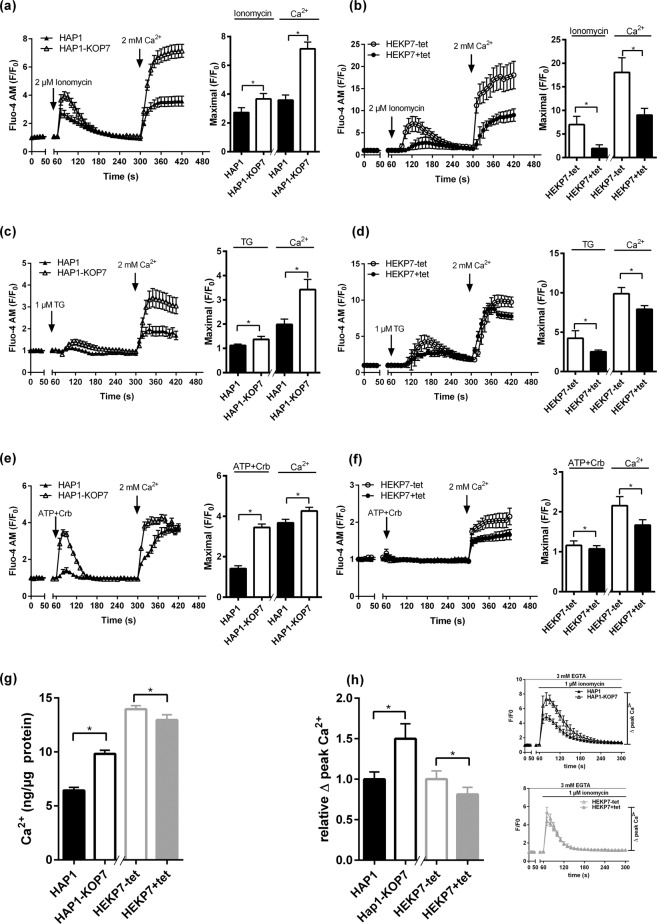
Ca^2+^ influx and Ca^2+^ stores in SLC10A7 overexpressing and SLC10A7 knockout cells. Calcium imaging was performed in HAP1 (control), HAP1-KOP7 (SLC10A7 knockout), HEKP7-tet (control, cells without tetracycline treatment), and HEKP7+tet (SLC10A7 overexpression after tetracycline treatment) cells pre-loaded with 2 µM Fluo-4 AM. Cells were treated with 2 µM ionomycin (**a**,**b**), 1 µM TG (**c**,**d**), or 100 µM ATP+Crb (**e**,**f**) in the absence of extracellular calcium to allow ER depletion. Then, 2 mM Ca^2+^ were added to allow store-operated Ca^2+^ entry. Fluorescence recording was performed every 10 s, and cell-based fluorescence was determined at defined regions of interest for each cell line (n = 6–10 for the HAP1 cells and n = 9–20 for the HEK293 cells), with a total number of about 84–260 cells. The bar graphs indicate the maximum peak data after ionomycin/TG/ATP+Crb (first peak) and calcium (second peak) treatment, respectively. (**g**) Cells were incubated in growth medium supplemented with 4 mM Ca^2+^ for 20 min. Total cellular Ca^2+^ was detected using a colorimetric calcium assay and was calculated after measuring the absorbance at 575 nm. All data were related to the total protein content of the cells. Data represent means ± SD of triplicate determinations of a representative experiment. (**h**) The Ca^2+^ content of intracellular stores was calculated by measuring the peak of ionomycin-mediated Ca^2+^ release in the presence of 3 mM extracellular EGTA (inset). Cell-based fluorescence was analyzed in each cell line at 10 defined regions of interest with a total number of about 180 to 260 cells. All data represent means ± SD of a representative experiment. *Significantly different with *p*  < 0.05 (Student’s t-test).

In both cell lines, calcium influx then was analyzed by fluorescence imaging after pre-loading the cells with the green-fluorescent calcium indicator Fluo-4 AM. After 1 min of background fluorescence recording, cells were treated with 2 µM ionomycin or 1 µM TG in the absence of extracellular calcium to allow calcium depletion of the calcium stores and activation of SOCE via the plasma membrane. In the HAP1-KOP7 cells, ionomycin and TG treatment resulted in a significant elevation of [Ca^2+^]_cyto_ compared with the wild type HAP1 cells. In both cell lines, calcium fluorescence then completely returned to baseline levels within 4 min (Fig. 2a,c). The activity in the SLC10A7-overexpressing cells was contrary to this. Here, SLC10A7 overexpression significantly reduced the calcium signals after ionomycin and TG treatment (Fig. 2b,d). This indicates that SLC10A7 expression is negatively correlated with the increase of [Ca^2+^]_cyto_ after ionomycin or TG treatment. The same was true after stimulation of the rapid calcium entry via SOCE after addition of 2 mM extracellular Ca^2+^. This calcium influx was about 2-fold higher in the HAP1-KOP7 cells compared to their HAP1 controls, but was significantly restricted by SLC10A7 overexpression in the HEKP7+tet cells (Fig. 2a–d). The addition of 2 mM extracellular Ca^2+^ in the absence of TG or ionomycin resulted in no difference in the calcium influx between all cell lines (Supplementary Fig. S1). In addition, the increase of [Ca^2+^]_cyto_ was analyzed after ATP + carbachol (Crb) treatment. Both compounds activate signaling cascades that deplete ER calcium stores via the inositol-tris-phosphate receptor (IP3R) pathway. Both cell lines were pre-incubated with 3 mM of the calcium chelator EGTA for 20 min prior to ATP+Crb treatment. In the HAP1-KOP7 cells, ATP+Crb treatment resulted in significantly higher increase of [Ca^2+^]_cyto_ compared to wild type HAP1 cells (Fig. 2e). In comparison, SLC10A7 overexpression significantly reduced the calcium signals after ATP+Crb treatment and the Ca^2+^ influx after the addition of extracellular Ca^2+^ (Fig. 2f). This data showed again that SLC10A7 expression is negatively correlated with the increase of [Ca^2+^]_cyto_.

To further clarify if the increased calcium fluorescence merely results from functionally higher influx rates or indeed reflects higher intracellular calcium contents, intracellular calcium was directly analyzed in the different cell lines under cultivation in normal medium supplemented with 4 mM Ca^2+^. As shown in Fig. 2g, the absence of SLC10A7 resulted in a significantly higher amount of calcium ions in HAP1-KOP7 cells compared to the HAP1 control cells. In contrast, SLC10A7 overexpression in HEKP7+tet cells significantly reduced the total amount of calcium ions compared to the HEKP7-tet controls. The calcium content of the intracellular organelles was also measured. For these experiments, the Ca^2+^ ionophore ionomycin was used, which is well known to rapidly release Ca^2+^ from internal Ca^2+^ stores. By additional application of the Ca^2+^ chelator EGTA to the incubation medium, Ca^2+^ influx from outside of the cell was prevented. Thereby, the calcium organelle content was estimated from the ionomycin-induced increase of [Ca^2+^]_cyto_. These calcium signals were significantly higher in HAP1-KOP7 cells compared to HAP1 control cells. In contrast, SLC10A7 overexpression in HEKP7+tet cells resulted in a significant reduction of the [Ca^2+^]_cyto_ increase (Fig. 2h).

### Overexpression of SLC10A7 inhibits SOCE in HAP1-KOP7 cells

**Figure 3:**
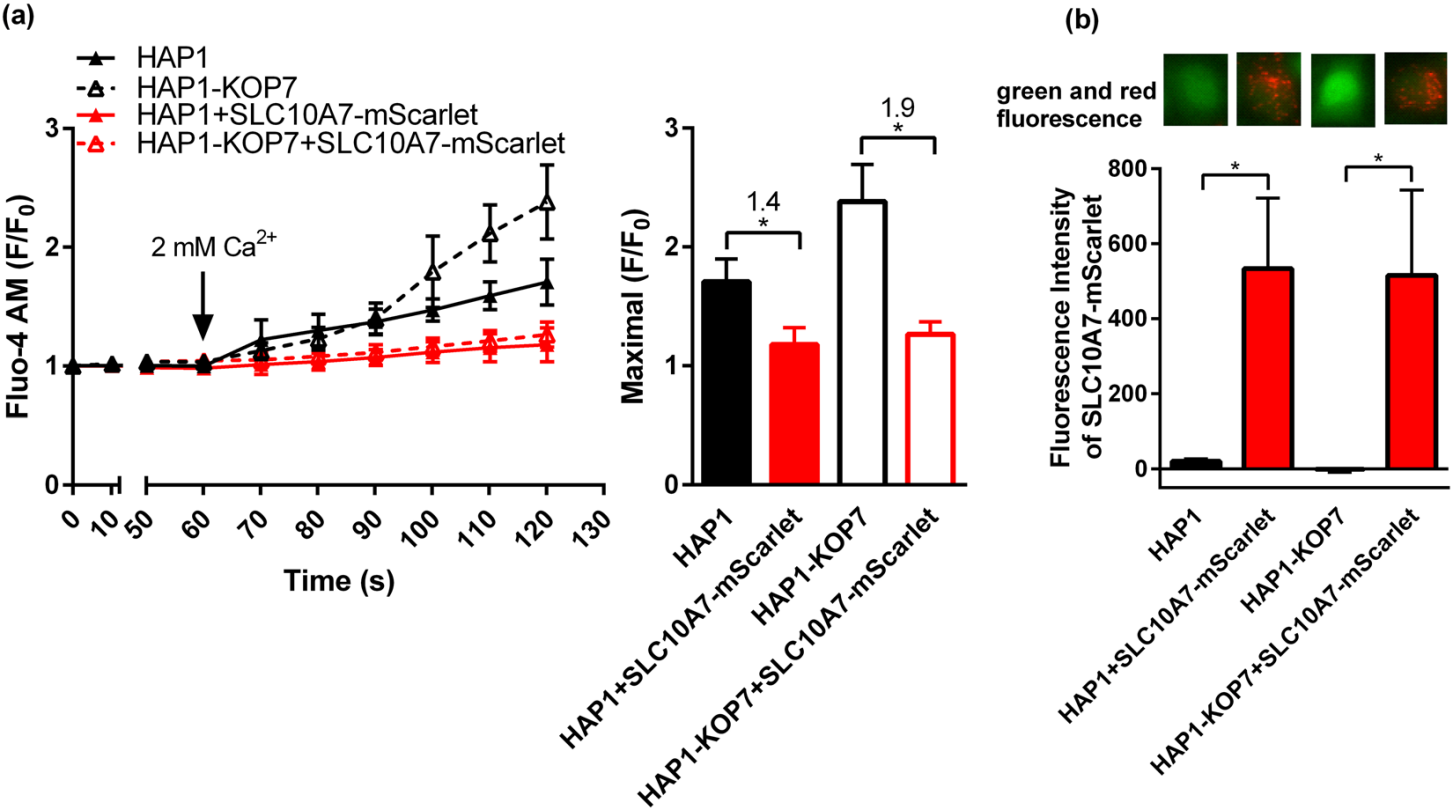
Complementation of the SLC10A7 knockout in HAP1-KOP7 cells. HAP1 and HAP1-KOP7 cells were transiently transfected with an SLC10A7-mScarlet construct (transcription variant v2), coding for the red fluorescent SLC10A7-mScarlet fusion protein. Then, cells were pre-loaded with 2 µM Fluo-4 AM and pre-treated with 1 µM TG and finally 2 mM extracellular Ca^2+^ were added. Red (mScarlet) and green (Fluo-4) fluorescence were recorded every 10 s in SLC10A7-mScarlet-expressing cells. The bar graphs represent the maximal induced calcium fluorescence (**a**) and the red fluorescence of the SLC10A7-mScarlet protein (**b**). (Images in **b**) Overlay of red and green fluorescence signals in representative individual cells. Fluorescence recording was performed every 10 s, and the fluorescence of 8–13 individual cells was determined for each cell line. All data represent means ± SD of a representative experiment. *Significantly different with *p*  < 0.05 (One-way ANOVA).

In order to determine if the different levels of calcium influx in HAP1 and HAP1-KOP7 cells truly result from different SLC10A7 expression, we transiently overexpressed an SLC10A7-mScarlet red fluorescent construct in the HAP1-KOP7 cells. Fluorescent labeling of SLC10A7 was of particular importance here, as it facilitated the selection of the SLC10A7-transfected cells for calcium imaging. As before (see Fig. 2c,d), all cells were pre-treated with 1 µM TG for 20 min to deplete intracellular calcium stores and to prevent Ca^2+^ reuptake into the ER. Then 2 mM extracellular Ca^2+^ was added, and calcium fluorescence was analyzed in the different cell lines, i.e. HAP1 (wild type), HAP1+SLC10A7-mScarlet (overexpression), HAP1-KOP7 (knockout), and HAP1-KOP7+SLC10A7-mScarlet (complementation of the knockout). As shown in Fig. 3a, calcium influx was negatively correlated with the SLC10A7 expression level, and was significantly higher in the HAP1-KOP7 cells compared to the HAP1 wild type cells. In both cell lines, the SLC10A7-mScarlet fluorescent protein was expressed at equal levels (Fig. 3b), and significantly suppressed calcium influx to comparable basic levels (Fig. 3a). Accordingly, in the SLC10A7-mScarlet overexpressing cells, only a few calcium signals (green fluorescence) were visible, whereas HAP1-KOP7 cells showed strong calcium fluorescence, and HAP1 cells revealed moderate green fluorescence (inset in Fig. 3b).

### SOCE inhibition affects intracellular Ca^2+^ release and Ca^2+^ influx

**Figure 4:**
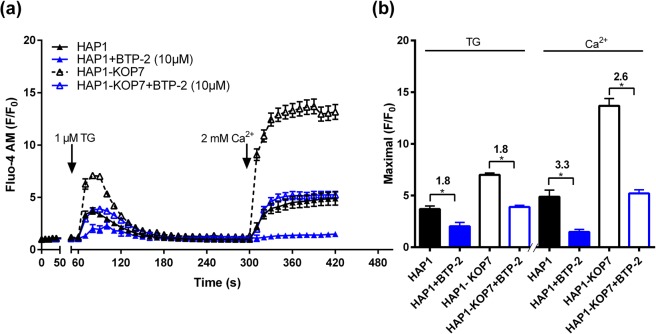
BTP-2 blocks intracellular calcium mobilization and calcium entry in HAP1 and HAP1-KOP7 cells. (**a**) HAP1 and HAP1-KOP7 cells were pre-loaded with 2 µM Fluo-4 AM and pre-treated with 10 µM BTP-2 or vehicle (DMSO) for 20 min. Then, 1 µM TG was added in the absence of extracellular calcium to allow ER depletion. 4 min later, 2 mM Ca^2+^ were added to allow store-operated Ca^2+^ entry. Fluorescence recording was performed every 10 s, and cell-based fluorescence was determined at six defined regions of interest for each cell line with a total number of about 90–100 cells. (**b**) The bar graph indicates the maximal peak data on calcium release from the ER (TG, first peak) and calcium entry via SOCE (Ca^2+^, second peak). All data represent means ± SD of a representative experiment. *Significantly different with *p* < 0.05 (One-way ANOVA).

Since we expected that SLC10A7 would regulate the calcium influx via inhibition of SOCE, we investigated the effect of the SOCE-inhibitor BTP-2 on the TG-induced and Ca^2+^-stimulated calcium fluorescence. HAP1 and HAP1-KOP7 cells were again loaded with Fluo-4 AM and pre-treated with 10 µM BTP-2 for 20 min. Then cells were treated with TG in the absence of extracellular calcium to allow depletion of intracellular calcium stores and activation of SOCE in the plasma membrane. As already shown, calcium fluorescence was significantly increased in the HAP1-KOP7 compared to the HAP1 cells, after both 1 µM TG and 2 mM Ca^2+^ incubation. Interestingly, BTP-2 significantly reduced the calcium fluorescence in both cell lines about 2-fold for the TG response and about 3-fold for the treatment with 2 mM Ca^2+^ (Fig. 4). BTP-2 reduced the calcium influx to baseline levels in the HAP1 cells, whereas in HAP1-KOP7, the calcium influx was proportionally lower, but still showed considerable fluorescence signals, comparable to those of the wild type HAP1 cells without BTP-2 inhibition.

### SLC10A7 co-localization with STIM1, Orai1 and SERCA2

**Figure 5:**
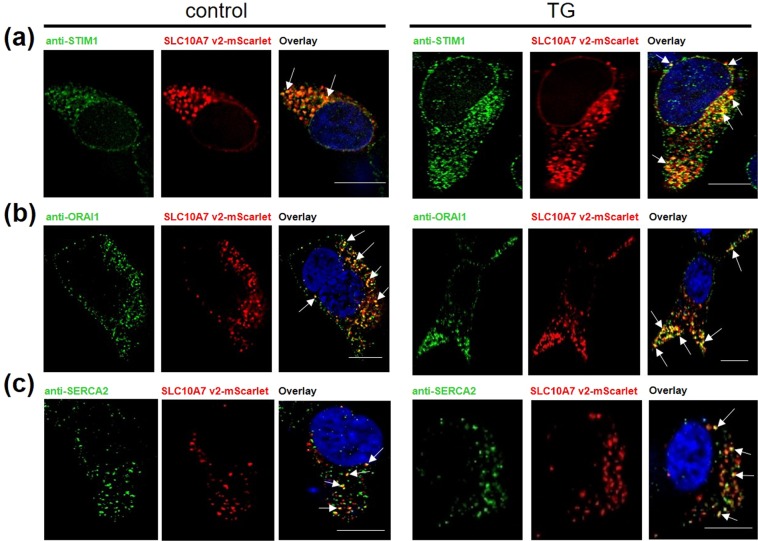
Co-localization of SLC10A7-mScarlet with STIM1, Orai1 and SERCA2 in HEK293 cells. HEK293 cells were seeded on coverslips and were transiently transfected with the SLC10A7-mScarlet (transcript variant v2) construct (red fluorescence). After 48 h, the cells were treated with 1 µM TG for 5 min and then the cellular co-localization with STIM1, Orai1 and SERCA2 was detected by immunofluorescence with respective anti-STIM1, anti-Orai1 and anti-SERCA2 antibodies (green fluorescence). Nuclei were stained with Hoechst 33342 (blue). Images represent maximum projections of z-stacks at 630-x magnification after deconvolution. Scale bars: 10 µm. Arrows: Co-localization of both proteins.

In order to localize the site of interaction of SLC10A7 with calcium signaling, we performed co-localization studies using the SLC10A7-mScarlet construct, transiently transfected into HEK293 cells, with STIM1, Orai1 and SERCA2. Interestingly, SLC10A7-mScarlet co-localized with all three proteins, STIM1 (Fig. 5a), Orai1 (Fig. 5b), and SERCA2 (Fig. 5c). Furthermore, we found a trend for a higher degree of co-localization between SLC10A7-mScarlet (red) and STIM1 (green) after TG treatment (Fig. 5a).

### SLC10A7 transcript variants v2 and v4 were expressed in most human tissues

**Figure 6:**
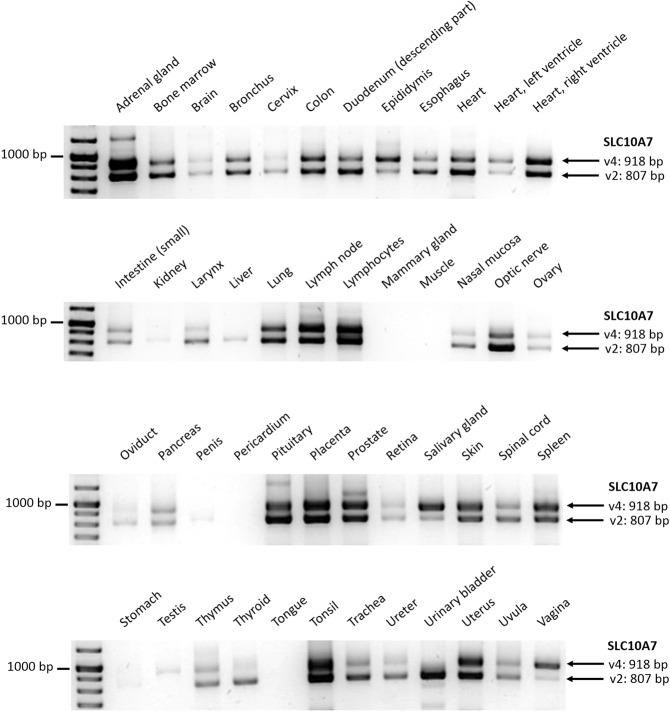
Expression pattern of SLC10A7 transcript variants v2 and v4 in different human tissues. Expression analysis was performed on a commercial major human tissue cDNA panel with primers theoretically allowing amplification of all different transcript variants (see Supplementary Table S1). Primers were designed to bind at exon boundary 3/4 (forward) and at exon 12 (reverse). Amplicons were separated on a 2.5% agarose gel. Only transcript variants v2 (amplicon size: 807 bp) and v4 (amplicon size: 918 bp) were detected. Individual bands were excised from the gel and sequence verified by DNA sequencing. Images were cropped from different gels for better clarity. The full-length gels are depicted in Supplementary Fig. 4.

**Figure 7:**
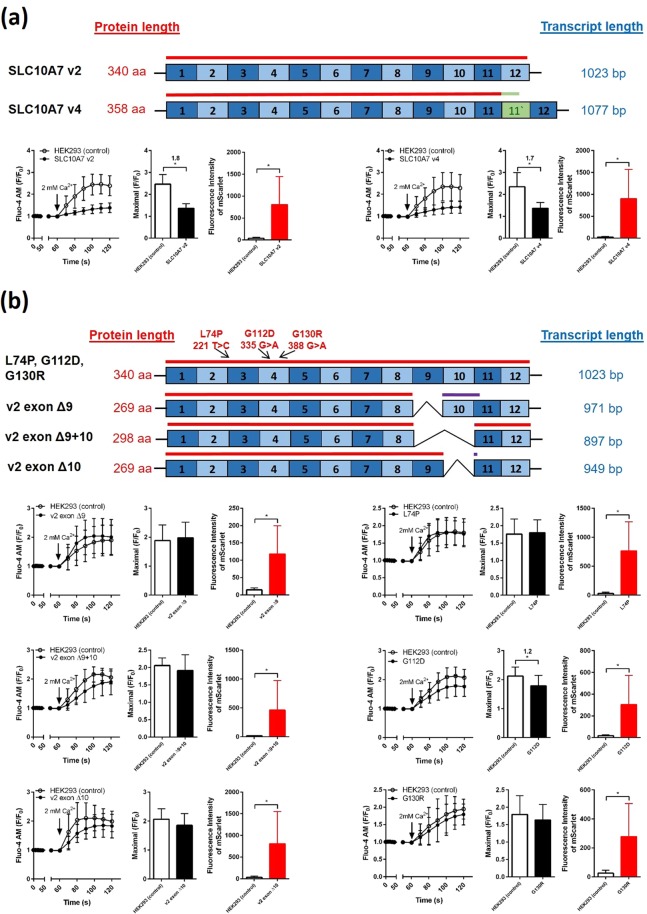
Effects of SLC10A7 transcript variants v2 and v4 as well as of different mutants on the Ca^2+^ influx into HEK293 cells. For each of the indicated SLC10A7 transcript variants (**a**) and mutants (**b**), respective SLC10A7-mScarlet constructs were generated and transiently transfected into HEK293 cells. At 48 hours after transfection, cells were prepared for calcium imaging by Fluo-4 AM (2 µM) and TG (1 µM) pre-incubation, followed by the addition of 2 mM extracellular Ca^2+^. Red (mScarlet) and green (Fluo-4) fluorescence signals were recorded every 10 s. Fluo-4 AM fluorescence signals then were separately analyzed in additionally red fluorescent cells (considered as SLC10A7-mScarlet expressing) and non-red fluorescent cells (un-transfected controls). The left bar graphs represent the maximal induced calcium fluorescence in both cell types and the right bar graphs indicate the fluorescence intensities of the SLC10A7-mScarlet fusion proteins. Data are means ± SD of 33–92 individual cells from representative experiments. *Significantly different with *p*  < 0.05 (Student’s t-test).

In order to analyze expression of SLC10A7 transcript variants in different human tissues, PCR primers were selected that allowed the amplification of known SLC10A7 variants at different amplicon lengths in a single PCR reaction. As shown in Fig. 6, SLC10A7 revealed a broad expression pattern, but transcript variant occurrence was not identical in all organs. SLC10A7 transcript variants 2 and 4 were amplified at comparable levels in most tissue cDNAs. Only few organs revealed dominant occurrence of transcript variant v2 (e.g. the urinary bladder), or SLC10A7 transcript variant v4 (e.g. salivary gland). Both variants only differ in their last few amino acids at the C-terminus, which is localized intracellularly (see Fig. 7a and Supplementary Figs. S2 and S3). Other SLC10A7 transcript variants listed in the GenBank database (being v1, v3, and v5, see Supplementary Table S1) were not detected at the mRNA level.

### Effects of SLC10A7 transcript variants and mutants on Ca^2+^ influx

All experiments involving overexpression of the SLC10A7 protein in HEK293 cells or complementation of the knockout in HAP1-KOP7 cells with the SLC10A7-mScarlet construct were performed with the SLC10A7 splice variant 2 (SLC10A7 v2) that we consider to be the physiologically most relevant coding sequence for the full-length SLC10A7 protein of 340 amino acids (Supplementary Fig. S2). However, as already described in a previous study, expression of the *SLC10A7* gene produces more than one SLC10A7 transcript variant^19^. However, based on the expression analysis shown in Fig. 6, only transcript variants v2 and v4 were considered relevant (see Supplementary Table S1, Supplementary Figs. S2 and S3, and Fig. 7a). In order to analyze if the SLC10A7 transcript variant v4 is also active in limiting SOCE via the plasma membrane, as it was demonstrated for transcript variant SLC10A7 v2 (see Fig. 2), SLC10A7-mScarlet tagged constructs for v2 and v4 were transiently transfected into HEK293 cells. Then calcium influx following TG treatment was analyzed after addition of 2 mM extracellular Ca^2+^. As shown in Fig. 7a, expression of the transcript variants SLC10A7-mScarlet v2 and v4 resulted in comparably significant inhibition of the calcium influx. Apart from the SLC10A7 transcript variants, SLC10A7 mutations previously described to be associated with human pathologies were functionally analyzed in HEK293 cells. These include the point mutations L74P, G112D, and G130R as well as the exon-skipping variants Δ9, Δ9+10, and Δ10 that were described in patients, due to genomic splice site mutations (see Supplementary Table S2). Different mutant SLC10A7-mScarlet constructs were generated as indicated in Fig. 7b, and were transiently transfected into HEK293 cells. All constructs revealed expression of the red fluorescent SLC10A7-mScarlet protein, as indicated by the red bars. However, none of mutant constructs analyzed revealed an inhibitory effect on calcium influx, as was shown for the wild type SLC10A7 protein. The only exception was the SLC10A7 variant G112D mutation, which showed a slightly but significantly reduced calcium influx compared to the non-SLC10A7-mScarlet expressing control cells (Fig. 7b).

## Discussion

In the present study, we provided evidence for the first time of a direct role of the human SLC10A7 protein in intracellular calcium signaling. Initially, SLC10A7 was thought to be a putative novel bile acid transporter, based on a certain sequence homology to members of the bile acid transporter family SLC10 (Solute Carrier Family 10)^19–21^. However, heterologous expression of the SLC10A7 protein in HEK293 cells and *Xenopus laevis* oocytes failed to show any transport activity for bile acids^19^. As SLC10A7 homologous proteins also exist in yeasts, bacteria and plants, we previously aimed to use one of these organisms to elucidate the function of the SLC10A7 protein. Therefore, SLC10A7 mutants of the yeast fungus *Candida albicans* were generated^22^. Interestingly, these mutants were hypersensitive to high concentrations of extracellular calcium and revealed increased calcium influx and cytosolic calcium levels, finally leading to the denomination of the SLC10A7-homologous protein in *Candida albicans* as regulator of calcium homeostasis CaRch1p^22,23^. Later, a functional homolog of CaRch1p was also identified in *Saccharomyces cerevisiae* (ScRch1p)^24^. This was the first step in establishing the role of Rch1p/SLC10A7 for calcium homeostasis. However, the precise cellular and molecular function of the human SLC10A7 protein still remained elusive and, therefore, in the present study, we aimed to clarify the functional role of human SLC10A7 and to verify if SLC10A7 patient mutations indeed reveal an SLC10A7 loss-of-function phenotype.

The basic finding of the present study is that increasing or decreasing expression levels of the human SLC10A7 protein lead to contrary effects on intracellular calcium levels and calcium signaling. Furthermore, SLC10A7 protein expression was negatively correlated with the store-operated calcium entry (SOCE). These findings overall indicate that SLC10A7 plays a role in regulation of cellular calcium homeostasis. Interestingly, the higher calcium levels and calcium influx into HAP1 cells lacking SLC10A7 expression phenocopy quite well the calcium sensitive phenotype of the Rch1 mutants of *Candida albicans* and *Saccharomyces cerevisiae*^22–24^. Furthermore, fibroblasts from SLC10A7-mutant patients also showed increased calcium influx compared to control fibroblasts^17^.

Ionomycin is a calcium ionophore that allows calcium release from the ER^25^ and enables Ca^2+^ influx through ionophore pores in the plasma membrane^26^. ATP and Crb activate signaling cascades that induce IP3R-mediated Ca^2+^ release from the ER via binding to respective G-protein-coupled receptors at the plasma membrane^15,27^. TG blocks SERCA that normally sequesters calcium in the SR/ER lumen^10,12,28^. All these compounds induced higher calcium signals in the HAP1 cells lacking SLC10A7 compared to wild type HAP1 cells. Although ionomycin, TG, and ATP+Crb are commonly used to induce calcium depletion from the ER^15,29–35^, we cannot completely reject that calcium release from mitochondria also takes place under these experimental conditions (see below). Therefore, further studies are needed to directly measure [Ca^2+^]_ER_ levels in HAP1 and HAP1-KOP7 cells^36^.

**Figure 8:**
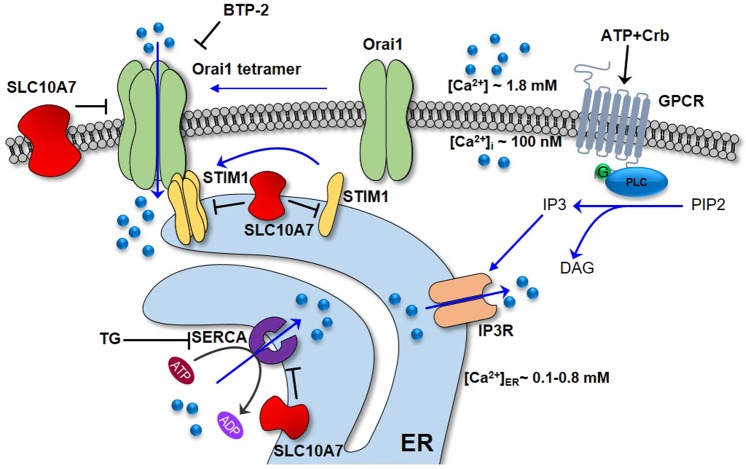
Schematic illustration of calcium signaling in non-excitable cells. Following the stimulation of G protein-coupled receptors (GPCR), phospholipase C (PLC) hydrolyses PIP2 to IP3. IP3 activates the IP3 receptor (IP3R) and triggers the depletion of ER Ca^2+^ stores. TG or ionomycin also cause the Ca^2+^ depletion from ER by different mechanisms. Declined ER Ca^2+^ levels are sensed by STIM1 proteins. STIM1 oligomerizes, migrates towards subplasmalemmal ER-PM junctions, and interacts with Orai1 to trigger store-operated calcium channel opening. Then, SERCA pumps Ca^2+^ back into the ER to refill the stores with Ca^2+^. SLC10A7 is hypothesized to negatively regulate STIM1, Orai1 and/or SERCA2 through protein interaction. TG, SERCA inhibitor; BTP-2, SOCE blocker.

How does SLC10A7 influence cellular calcium levels and calcium signaling on the molecular level? There are several possible explanations (see Fig. 8). SLC10A7 might limit the transport capacity of SERCA or might increase the rate of Ca^2+^ leaking from the ER, so that in the SLC10A7 knockout cells the storage of calcium in the ER is increased. In addition, SLC10A7 might negatively regulate STIM1 and/or Orai1, e.g. by affecting the sensitivity of STIM1 to calcium, or decrease the probability of opening Orai1. A role of SLC10A7 in STIM1-Orai1 complex formation or its stability at the plasma membrane is also possible. Consequently, activation of SOCE via the STIM-Orai complex might be more pronounced due to the higher amplitude of ER calcium depletion after TG, ionomycin, or ATP+Crb treatment. Interestingly, clear co-localization of SLC10A7 with all three proteins, SERCA, STIM1, and Orai-1 was observed by immunofluorescence microscopy. However, any potential direct protein-protein interaction of these proteins and the exact mechanism of such an interaction have to be further investigated.

So far, several regulator proteins of the STIM-Orai complex and of SERCA have been described. These include CRACR2A that interacts with the N-terminus of Orai1 and functions as a Ca^2+^ sensor in the cytoplasm. Immunoprecipitation and microscopy studies revealed that CRACR2A showed clustering with STIM1 and Orai1 in T-cells, and showed partial co-localization with STIM1 without store depletion in HEK293 cells^13^. In addition, knockout of CRACR2A decreased TG-induced SOCE in HEK293 and Jurkat T cells, and, conversely, overexpression of CRACR2A increased TG-induced SOCE in HeLa and Jurkat T cells^13^. Another regulator is SARAF, which has been characterized as a negative regulator of SOCE through its interaction with STIM1^15^. Like SLC10A7, overexpression or knockout of SARAF expression resulted in opposite effects on intracellular Ca^2+^ levels. SARAF was localized in the ER and co-localized with STIM1, but SARAF expression was also detected in the plasma membrane of SH-SY5Y neuroblastoma cells^37^. Based on this, it is also possible that SLC10A7 mediates its effects on the calcium signaling indirectly via CRACR2A or SARAF. In the case of SERCA, phospholamban and sarcolipin have been identified as regulatory factors, and both of them co-localize with SERCA in muscle cells. While binding of phospholamban lowers SERCA pump affinity for Ca^2+^, sarcolipin decreases the V_max_ of SERCA Ca^2+^ transport^11,38–40^. In a similar manner as phospholamban and sarcolipin, SLC10A7 might negatively regulate SERCA in non-muscle cells, so that in the absence of SLC10A7, SERCA becomes more active in sequestering calcium in the ER.

Apart from the discussed effects of SLC10A7 on STIM1, Orai1 and SERCA, an effect of SLC10A7 on cellular Ca^2+^ buffering, to which mitochondria contribute essentially^41^, cannot be excluded. If, for example, SLC10A7 stimulated the mitochondrial Ca^2+^ uniporter (MCU) responsible for exchange of Ca^2+^ between the cytosol and mitochondria^42^, the changes in the cytosolic Ca^2+^ concentration measured with Fluo-4 would also be enhanced after SLC10A7 knockout. However, such a mechanism would not be able to explain the increase in total cellular Ca^2+^ amount after SLC10A7 knockout, or its decrease after upregulation of SLC10A7 (Fig. 2g), as any change in MCU activity would only affect cellular Ca^2+^ distribution, but not the overall content of Ca^2+^. Thus, an interaction of SLC10A7 with Ca^2+^ buffering seems to be unlikely as explanation for the results obtained in the present study.

How is the proposed regulatory function of SLC10A7 linked to the clinical phenotype of patients with *SLC10A7* mutations? These patients typically show skeletal dysplasia, amelogenesis imperfecta, and decreased bone mineral density with differences in the severity of the phenotype depending on the exact site of mutation^16–18^. On the molecular level, altered glycosaminoglycan synthesis, intracellular mis-localization of glycoproteins and defective post-Golgi transport of glycoproteins to the extracellular matrix, as well as defective bone/enamel mineralization were described. While alterations in the composition of the extracellular matrix may have caused growth plate disorganization, growth delay, and skeletal dysplasia in the patients, the hypo-mineralization might be responsible for the amelogenesis imperfecta phenotype. While Ashikov *et al*. (2018) identified SLC10A7 mutant patients from a cohort of patients with abnormal Golgi glycosylation^16^, in the studies of Dubail *et al*. (2018) and Laugel-Haushalter *et al*. (2019), patients clinically presented with skeletal dysplasia and amelogenesis imperfecta^17,18^. All patients then underwent whole-exome sequencing and so the different *SLC10A7* mutations were identified. It is already known that synthesis and secretion of proteoglycans is dependent on the ER and Golgi calcium concentration^43^. This process might be disturbed by the significant effect of SLC10A7 on calcium signaling and [Ca^2+^]_ER_. On the other hand, it is interesting to note that patients with loss-of-function mutations in STIM1 and Orai1 also display an amelogenesis imperfecta phenotype, indicating that dysregulation of SOCE has a direct effect on bone/enamel mineralization^44–48^. Based on this, it can be suggested that dysregulation of calcium homoeostasis by human *SLC10A7* mutation represents the initial defect that subsequently leads to impaired glycosaminoglycan synthesis, disturbed glycoprotein transport, and bone/enamel hypo-mineralization, and finally ends in the clinical phenotype of skeletal dysplasia, amelogenesis imperfecta, and decreased bone mineral density^16–18^. However, the direct role of SLC10A7 for glycosaminoglycan synthesis, glycoprotein trafficking and bone/enamel mineralization has to be further investigated using appropriate cell culture models, such as HAP1 and HAP1-KOP7, and *Slc10a7*^*−/−*^ knockout mice.

In conclusion, the present study characterized for the first time the molecular function of the orphan carrier SLC10A7, which was established as a novel negative regulator of intracellular calcium signaling that most likely acts via STIM1, Orai1 and/or SERCA2 inhibition. Based on this, SLC10A7 is suggested to be named as negative regulator of intracellular calcium signaling (in short: RCAS). The fact that the protein function of SLC10A7 now can be measured as a function of the TG-induced calcium influx enabled us to analyze if the *SLC10A7* genomic mutations previously identified in patients with skeletal dysplasia, amelogenesis imperfecta and decreased bone mineral density indeed hamper the function of the coded protein. Indeed, all analyzed SLC10A7 mutants, except of G112D, showed a complete loss of function. This now provides the basis to further clarify the pathogenesis of these SLC10A7-associated diseases.

## Methods

### Materials

All of the chemicals, unless otherwise stated, were from Sigma-Aldrich (Taufkirchen, Germany), including thapsigargin (TG, T9033), ATP (A6144) and carbachol (212385M). Fluo-4 AM (F14201) and BTP-2 (*N*-[4-[3,5-bis(trifluoromethyl)pyrazol-1-yl]phenyl]-4-methylthiadiazole-5-carboxamide, 203890) were purchased from Thermo Fisher Scientific and Merck, respectively. Ca^2+^-free HEPES buffer was prepared as follows: NaCl 140 mM, KCl 4 mM, Hepes 10 mM, MgCl_2_ 1 mM, and glucose 25 mM; pH 7.4.

### Cell culture

HEK293 cells were maintained in DMEM/F12 medium (Gibco, Carlsbad, CA, USA) supplemented with 10% fetal calf serum (FCS, Pan-Biotech, Aidenbach, Germany), L-glutamine (4 mM, Anprotec, Bruckburg, Germany), penicillin (100 U/ml, Anprotec), and streptomycin (100 µg/ml, Anprotec) at 37 °C, 5% CO_2_, and 95% humidity. HAP1 represents a near-haploid human cell line that was derived from the male chronic myelogenous leukemia cell line KBM-7 cells and was purchased from Horizon Genomics (Cambridge, UK). The HAP1 SLC10A7 knockout cell line (further referred to as HAP1-KOP7) was engineered by CRISPR/Cas9 mutation and features a genomic 23-bp deletion in coding exon 2 of the *SLC10A7* gene (clone HZGHC005272c010, Horizon). HAP1 cells were cultured in Iscove’s Modified Dulbecco’s Medium (IMDM) (Thermo Fisher Scientific, Dreieich, Germany).

### Stable transfection of HEK293 cells

The recombinant human cell line SLC10A7-HEK293 (further referred to as HEKP7) was generated based on Flp-In T-REx 293 cells (Thermo Fisher Scientific), as described previously^21^. The full-length SLC10A7 open reading frame (according to GenBank accession number NM_001029998.6, transcript variant 2) was cloned into the pcDNA5/FRT/TO expression vector (Invitrogen), and was used for stable transfection of Flp-In T-REx-293 cells. Flp-In T-Rex-293 cells contain a tetracycline-regulated CMV/tetO2 hybrid promoter that allows expression of the gene of interest only under tetracycline (tet) treatment.

### Construction of the mScarlet tagged SLC10A7 constructs

For SLC10A7 transcription variants v2 and v4, as well as for all known SLC10A7 mutants (listed in Supplementary Tables S1 and S2), C-terminally mScarlet tagged constructs were generated as reported before^49^. Briefly, the flexible linker protein sequence GGGGSGGGGSGGGG, followed by the cDNA sequence coding for the monomeric red fluorescent protein mScarlet^50^ were added to all constructs via DNASTAR 15.0 SeqBuilder Pro and were synthesized by Biocat (Heidelberg, Germany) into the pcDNA3.1(+) expression vector (Thermo Fisher Scientific).

### Transient transfection of HEK293 cells

HEK293 were transfected using Lipofectamine 2000 (Thermo Fisher Scientific) according to the manufacturer’s instructions. Briefly, 40 ×10^3^ cells were plated onto µ-Slide 8-well coverslips (IBIDI, Gräfelfing, Germany). The following day, cells were transfected with a total of 0.25 µg SLC10A7-mScarlet plasmid DNA by Lipofectamine 2000. The transfected cells were maintained in cultures for 48 h. Fluorescence was visualized on a Leica DM5500 fluorescence microscope (Leica, Wetzlar, Germany). Images were analyzed with the Leica Fluorescence Workstation software LAS-X.

### Sequencing analysis of SLC10A7

Genomic DNA from HAP1 and HAP1-KOP7 cells was isolated using the QIAamp DNA Mini Kit (Qiagen, Hilden, Germany) and was used for PCR amplification with the following oligonucleotide primers: 5′-TAG GAA TGA AAC ACA AGT CCT TTG C-3′ forward and 5′-ACA AAT AGA TTC TTC TTT TGT GCC A-3’ reverse. PCR products were separated on a 1.5% agarose gel, stained with GelRed (Biotium, Fremont, USA), and visualized on a UV-Transilluminator (ImageMaster, Pharmacia Biotech, Uppsala, Sweden). Relevant amplicons were excised under UV light and extracted with Hi Yield Gel/PCR DNA Fragment Extraction Kit (SLG, Gauting, Germany) and were subjected to DNA sequencing (Seqlab Microsynth, Göttingen, Germany).

### PCR amplification of SLC10A7 transcript variants

The TissueScan Human Major Tissue Plate by OriGene Technologies (Lot#TH28) was used to determinate expression of SLC10A7 transcript variants in different human tissues. For PCR amplification, Phusion Flash High-Fidelity DNA Polymerase (Thermo Scientific), forward primer (5′-GGC TTT TAA AAG GTT TGC AGA CAG TAG G-3′), and reverse primer (5′-GAG GCA ACA TTC ACA AGT ACA AGT CTT CAG-3′) were added to the pre-spotted cDNA panel in each well. PCR amplification was performed on the Applied Biosystems 7300 Real-Time PCR System at 45 cycles under the following conditions: initialization for 90 s at 98 °C, denaturation for 30 s at 98 °C, annealing for 30 s at 64 °C, extension for 1 min at 72 °C, final elongation for 1 min at 72 °C and final hold at 4 °C. After amplification, PCR products were separated by 2.5% agarose gel electrophoresis in TAE-buffer. The gel was dyed for 45 min in GelRed solution. GeneRuler DNA Ladder Mix (Thermo Scientific) was used to determinate the size of the bands. Representative PCR amplicons were excised from the gel and sequence verified by DNA sequencing (Seqlab Microsynth).

### Real-time RT-PCR analysis

Cells were harvested, and total mRNA was extracted by Maxwell RSC simplyRNA Tissue Kit (Promega). Complementary cDNA was synthesized from 1 µg total RNA by using 8 µl of RT-mix SuperScript III Reverse Transcriptase (Invitrogen). For quantitative expression analysis of the SLC10A7 transcripts, the gene-specific *TaqMan Gene Expression Assay* Hs04397477_m1 (Thermo Fisher Scientific) was used. The assay Hs02758991_g1 (ThermoFisher) was used for control amplification of GAPDH. The plates were heated for 2 min at 50 °C, 10 min at 95 °C, and then 40 cycles of 15 s at 94 °C and 60 s at 60 °C were applied. All data were expressed as fold changes using the 2^−ΔΔCt^ method.

### Quantification of the SLC10A7 protein in the HEK293 and HAP1 cell lines

Protein abundance of ABCB1 (P-gp), ABCC1 (MRP1) and ABCC2 (MRP2) and SLC10A7 were analyzed by mass spectrometry (MS)-based targeted proteomics using validated LC−MS/MS methods^51^. In brief, pellets of HEK293 and HAP1 cells were lysed and the membrane protein fraction was extracted using the ProteoExtract Native Membrane Protein Extraction kit (Merck, Darmstadt, Germany) according to the manufacturer’s protocol. All sample preparation and digestion steps were performed using Protein LoBind tubes (Eppendorf, Hamburg, Germany). Protein quantification was conducted on a 5500 QTRAP triple quadrupole mass spectrometer (AB Sciex, Darmstadt, Germany) coupled to an Agilent Technologies 1260 Infinity system (Agilent Technologies). The following peptides were used for quantification of the respective proteins: ABCB1, AGAVAEEVLAAIR; ABCC1, DGAFAEFLR; ABCC2, LTIIPQDPILFSGSLR; and SLC10A7, TEELTSALVHLK. For each peptide, three mass-to-charge transitions were used for quantification (range: 0.1–25 nmol/L). Accuracy (error) and precision (CV) during sample analysis were both below 20%. Final protein abundance data (picomoles per milligram protein) were calculated by normalization to the total protein content of the isolated membrane fraction.

### Ca^2+^ imaging

Cells were plated at a density of 40 × 10^3^ cells per well in 8-well μ-slides (IBIDI) in culture medium containing 10% FCS. After 24 h, the medium was replaced by fresh serum-free medium for 2 h. Then, cells were incubated in Ca^2+^-free HEPES containing 2 µM Fluo-4 AM for 30 min at RT and were washed three times with Ca^2+^-free HEPES to remove any excess extracellular dye. Cells were incubated for an additional 30 min to allow complete de-esterification of intracellular AM esters. The plates were set under the DM5500 Leica fluorescent microscope and basal fluorescence was recorded for 1 min. Calcium-induced fluorescence was recorded by the LAS-X imaging software and cell-based fluorescence was determined by defined regions of interest (ROI). Data are presented as the mean background-subtracted fluorescence intensity of each cell, normalized to the intensity of the first image (F/F0).

### Measurement of intracellular calcium ion content

Cells were plated onto 15 cm^2^ petri dishes and were treated with 4 mM CaCl_2_ for 20 min. After treatment, the cells were washed with phosphate-buffered saline (PBS, 137 mM NaCl, 2.7 mM KCl, 1.5 mM KH_2_PO_4_, 7.3 mM Na_2_HPO_4_, pH 7.4) and the scraped cells were centrifuged at 1,000 rpm for 2 min and then were immediately placed on ice. Measurement of the calcium ion content was performed using the Calcium Colorimetric Assay (MAK022) in 96-well plates at the GloMax Multi Detection System (Promega). The total protein content of the samples was determined using the BCA protein assay kit (Merck).

### Immunofluorescence

HEK293 cells transiently transfected with the SLC10A7-mScarlet construct were grown on poly-L-lysine coated 8-well µ-slide (IBIDI). After 48 h, cells were incubated with or without 1 µM TG for 5 min and fixed with 2% PFA and blocked with blocking buffer (containing 1% bovine serum albumin and 4% goat serum in PBS) for 30 min at room temperature. Then, cells were incubated at 4 °C overnight with antibodies against STIM1 (1:800, D99E10, Cell Signaling), Orai1 (2 µg/ml, sc-377281, Santa Cruz Biotechnology, Heidelberg, Germany) or SERCA2 (1:250, sc-376235, Santa Cruz Biotechnology) in blocking buffer, followed by labeling with AlexaFluor 488-conjugated secondary antibodies (Invitrogen) and nuclear marker Hoechst 33342. Z-stack cell imaging was performed at room temperature on an inverted Leica DM5500 fluorescence microscope.

### Data and statistical analyses

Statistical analysis was performed by using Student’s t-test or one-way ANOVA with GraphPad Prism 6.0 (GraphPad Software Inc., San Diego, CA, USA). Error bars represent mean ± SD. The numbers of samples and experimental repetitions are indicated in the figure legends. A level of *p* < 0.05 was considered as statistically significant.

## Supplementary information

Supplementary information.
