# Supplementary material for: The orphan solute carrier SLC10A7 is a novel negative regulator of intracellular calcium signaling
Source: Sci Rep. 2020 Apr 29;10:7248. doi: 10.1038/s41598-020-64006-3 (PMC7190670; doi:10.1038/s41598-020-64006-3)
Supplement: Supplementary file 1 — Supplementary information. [file 41598_2020_64006_MOESM1_ESM.pdf]

The orphan solute carrier SLC10A7 is a novel negative regulator of intracellular calcium signaling

Emre Karakus[1], Marie Wannowius[1], Simon Franz Müller[1], Silke Leiting[1], Regina Leidolf[1], Saskia Noppes[1], Stefan Oswald[2], Martin Diener[3], Joachim Geyer[1*]

[1]*Institute of Pharmacology and Toxicology, Faculty of Veterinary Medicine, Justus Liebig University Giessen, 35392 Giessen, Germany*

[2]*Institute of Pharmacology, University of Greifswald, 17487 Greifswald, Germany*

[3]*Institute of Veterinary Physiology and Biochemistry, Faculty of Veterinary Medicine, Justus Liebig University Giessen, 35392 Giessen, Germany*

Supplementary Table S1. SLC10A7 transcript variants

Supplementary Table S2. SLC10A7 mutants

Supplementary Fig. S1. Ca^{2+} influx into SLC10A7 knockout and overexpressing cells

Supplementary Fig. S2. SLC10A7 transcript variant v2

Supplementary Fig. S3. SLC10A7 transcript variant v4

Supplementary Fig. S4. Full-length gels corresponding to Fig. 6.

SLC10A7 transcript variant	Transcript length	CDS (ORF length)	GenBank Accession No.	Protein isoform	Protein length	GenBank Accession No.	Comment
V1	805 bp	1...561 (561 bp)	NM_001030316	a	186 aa	NP_001025487	Δexon8+9, off-frame, premature stop codon, nonsense-mediated mRNA decay (NMD) candidate
V2	3756 bp	224...1246 (1023 bp)	NM_001029998	b	340 aa	NP_001025169	12 coding exons, reference sequence
V3	1821 bp	255-734 (480 bp)	NM_032128	c	159 aa	NP_115504	Coding exons: 1-4 and additional exon 4'
V4	3867 bp	224...1300 (1077 bp)	NM_001300842	d	358 aa	NP_001287771	Additional exon 11' between exon 11 and exon 12, alternative C-terminus compared with isoform b
V5	3717 bp	224...1207 (984 bp)	NM_001317816	e	327 aa	NP_001304745	Δexon5, in frame

Supplementary Table S1. SLC10A7 transcript variants.

Nucleotide change	mRNA change	Protein change	Reference
c.722-16A>G (intron 8)	Exon 9 skipping due to splice acceptor site mutation	Frameshift at p.Ile241 and premature stop codon at p.Ala270	1
c.335G>A (exon 4)		G112D in transmembrane domain 4	1
c.221T>C (exon 3)		L74P in transmembrane domain 3, reduced protein expression	2
c.388G>A (exon 4)		G130R in the second intracellular loop	2
c.773+1G>A (intron 9)	Exon 9 skipping due to splice donor site mutation	Frameshift at p.Ile241 and premature stop codon at p.Ala270	2
c.774-1G>A (intron 9)	Exon 10 skipping due to splice acceptor site mutation	Frameshift at p.Thr.258 and premature stop codon at p.Ala270	2
c.774-1G>A (intron 9)	Exon 9+10 skipping due to splice acceptor site mutation	In-frame deletion of 42 amino acids	
c.514C>T (exon 7)*		Premature stop codon at p.Gln172	2
c.908C>T (exon 11)*		P303L in transmembrane domain 10	3

*Not analyzed in the present study

Supplementary Table S2. SLC10A7 mutants.

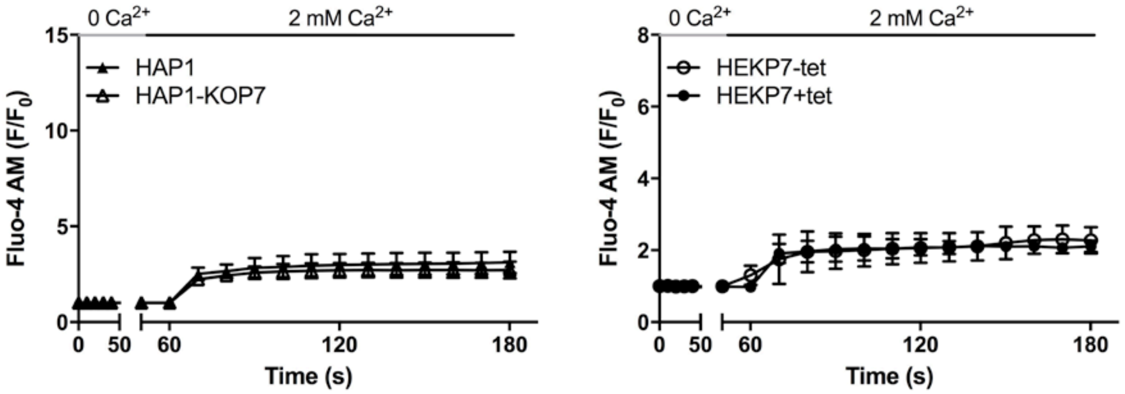

Supplementary Fig. S1. Ca^{2+} influx into SLC10A7 knockout and overexpressing cells. Calcium imaging was performed in HAP1 (control), HAP1-KOP7 (SLC10A7 knockout), HEKP7-tet (control, cells without tetracycline treatment), and HEKP7+tet (SLC10A7 overexpression) cells pre-loaded with 2 µM Fluo-4 AM. 2 mM Ca^{2+} were added and fluorescence recording was performed every 10 s. Cell-based fluorescence was determined at defined regions of interest (n=30 for the HAP1 cells and n=40 for the HEK293 cells) for each cell line with a total number of about 360-480 cells.

SLC10A7 transcript variant 2

(a)

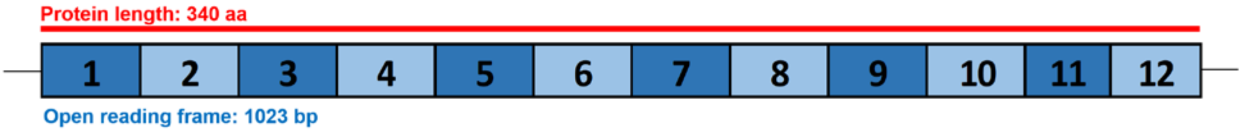

(b)

Exon	CDS (bp)	5`-Splice donor	3`-Splice acceptor	Intron size (kb)
1	100	ATGGGG/gtaagt	ttttag/GACCAC	4.4
2	83	ACAGAG/gtactg	ttccag/GAGCTG	6.9
3	137	AAAAGG/gtatgt	tttcag/TTTGCA	5.9
4	76	AATGAG/gtgagt	tcatag/GCAGCT	61.0
5	39	TTTTTG/gtaagt	tttcag/GGCATC	116.7
6	36	CTTTTT/gtgagt	ttacag/CTTGGT	19.9
7	84	GGACAG/gtaagg	tttcag/ATTGTC	11.8
8	166	TCATAA/gtaagt	cttcag/TATTTT	0.9
9	52	AACAAG/gtaagt	ccctag/GAATAA	9.6
10	74	CATTGG/gtaagt	caacag/GAATTC	24.3
11	146	CAGAAG/gtgagt	ttgtag/GGAGTG	2.1
12	30	GTATAA		

(c)

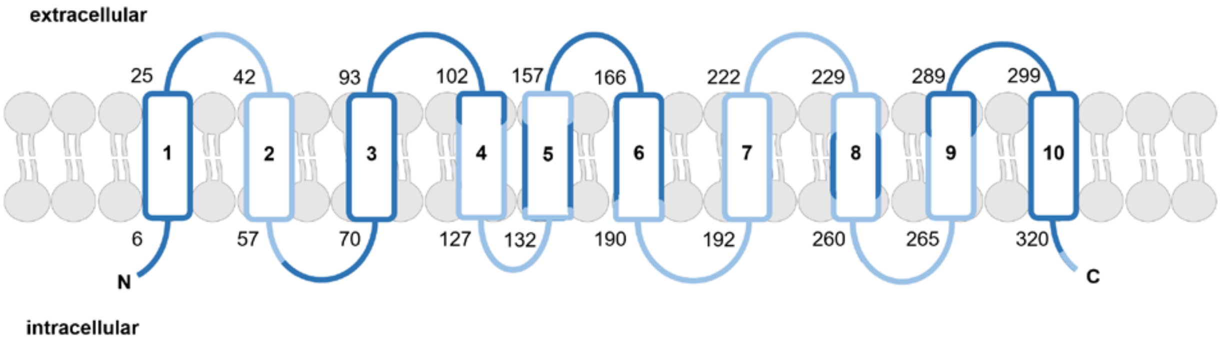

(d)

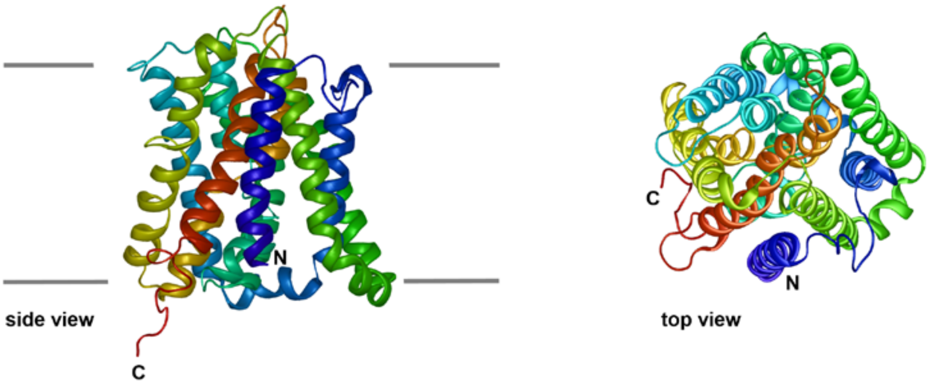

SLC10A7 transcript variant 2

(e)

```
Homo sapiens               1 MRLLERMRKDWFMVGIVLAIAGAKLEPSIGVNGGPLKPEITVSYIAVATIFFNSGLSLKTEELTSALVHLKLHLFIQI
Mus musculus               1 MRLLERARKEWFMVGIVVAIGAAKLEPSVGVNGGPLKPEITVSYIAVATIFFNSGLSLKTEELTSALVHLRLHLFIQI
Rattus norvegicus          1 MRLLERVRKEWFMVGIVVAIGAAKLEPSVGVNGGPLKPEITVSYIAVATIFFNSGLSLKTEELTSALVHLKLHLFIQV
Bos taurus                 1 MRLLERMRKEWFMIGIVLAIAGAKLEPSIGMNGGPLKPEITVSYIAVATIFFNSGLSLNTEELTSALVHIKLHLFIQI
Sus scrofa                 1 MRLLERIRKEWFMIGIVLAIAGAKLEPSIGVNGGPLKPEITVSYIAVATIFFNSGLSLNTEELTSALVHIKLHLFIQI
Equus caballus             1 MRLLERMRKEWFMVGIVLAIAGAKLEPSVGVNGGPLKPEITVSYIAVATIFFNSGLSLKTEELTSALVHIKLHLFIQI
Canis lupus familiaris     1 MRLLERMRKEWFMIGIVLAIAGAKLEPSVGVNGGPLKPEITVSYIAVATIFFNSGLSLKTEELTSALVHIKLHLFIQI
Felis catus                1 MRLLERMRKEWFMIGIVLAIAGAKLEPSIGVNGGPLKPEITVSYIAVATIFFNSGLSLKTEELTSALVHIKLHLFIQI
Gallus gallus              1 MGLLERLRKEWFIAGIALVIAAARLEPAVGVKGGPLKPEITITYIAVSAIFFNSGLSLKTEELTSALMHVKLHLFVQI
Danio rerio                1 MGLIARVRKEWFIIGIVLVITFAKLQPSVGVKGGPLHPEITITYVAVSVIFFNSGLSLKTEELASALMHVKLHFFVQT
Xenopus laevis             1 MGLLERLRKEWFIVGIILVIAAAKLEPTVGVKGGPLKPEITITYIAVSAIFFNSGLSLKTEELTNALMHVKLHLFVQL

Homo sapiens              79 FTLAFFPATIWLFLQLLSITPINEWLLKGLQTVGCMPPPVSSAVILTKAVGGNEAAAIFNSAFGSFLGIVITPLLLLL
Mus musculus              79 FTLAFFPAAIWLFLQLLSVTSINEWLLKGLQTVGCMPPPVSSAVILTKAVGGNEAAAIFNSAFGSFLGIVVTPVLLLL
Rattus norvegicus         79 FTLAFFPTTIWLFLQLLSVTSINEWLLKGLQTVGCMPPPVSSAVILTKAVGGNEAAAIFNSAFGSFLGIVVTPVLLLL
Bos taurus                79 FTLAFFPAAVWLFLQLLSITPINEWLLKGLQTVGCMPPPVSSAVILTKAVGGNEAAAIFNSAFGSFLGIVVTPLLLLL
Sus scrofa                79 FTLAFFPAAIWLFLQLLSITPINEWLLKGLQTVGCMPPPVSSAVILTKAVGGNEAAAIFNSAFGSFLGIVVTPLLLLL
Equus caballus            79 FTLAFFPAAIWLFLQLLSITPINEWLLKGLQTVGCMPPPVSSAVILTKAVGGNEAAAIFNSAFGSFLGIVITPLLLLL
Canis lupus familiaris    79 FTLAFFPATIWLFLQLLSITPINEWLLKGLQTVGCMPPPVSSAVILTKAVGGNEAAAIFNSAFGSFLGIIITPLLLLL
Felis catus               79 FTLAFFPATIWLFLQLLSITPINEWLLKGLQTVGCMPPPVSSAVILTKAVGGNEAAAIFNSAFGSFLGIVVTPLLLLL
Gallus gallus             79 FTLVFFPTAIWLFLQLLSITPINEWLLKGLQTVGCMPPPVSSAVILTKAVGGNEAAAIFNSAFGSFLGIVITPLLLLL
Danio rerio               79 FTLVFFPIAIWLLLKVLALTAINEWLLRGLQTVACMPPPVSSAVILTKAVGGNEAAAIFNSAFGSFLGIVVTPLLLLV
Xenopus laevis            79 FTLVFFPTAIWLFLQVLSLTPINEWLLKGLQTVSCMPPPVSSAVILTKAVGGNEAAAIFNSAFGSFLGIVVTPLLLLL

Homo sapiens             157 FLGSSSSVPFTSIFSQLFMTVVVPLIIGQIVRRYIKDWLERKKPPFGAISSSVLLMIIYTTFCDTFSNPNIDLDKFSL
Mus musculus             157 FLGSSSSVPFTSIFSQLFMTVVVPLVIGQIVRRYIKDWLERKKPPFGVVSSSVLLMIIYTTFCDTFSNPNIDLDKFSL
Rattus norvegicus        157 FLGSSSSVPFTSIFSQLFMTVVVPLVIGQIVRRYIKDWLERKKPPFGVVSSSVLLMIIYTTFCDTFSNPNIDLDKFSL
Bos taurus               157 FLGSSSSVPFTSIFSQLFMTVVVPLIIGQIVRRYIKDWLERKQPPFGAVSSSVLLMIIYTTFCDTFSNPSIDLDKFSL
Sus scrofa               157 FLGSSSSVPFTSIFSQLFMTVVVPLIIGQIVRRYIKDWLERRKPPFGAVSSSVLLMIIYTTFCDTFSNPNFDLDKFSL
Equus caballus           157 FLGSSSSVPFTSIFSQLFMTVVVPLIIGQIVRRYIKDWLERKKPPFGAISSSVLLMIIYTTFCDTFSNPNIDLDKFSL
Canis lupus familiaris   157 FLGSSSSVPFTSIFSQLFMTVVVPLIIGQIFRRYIKDWLERKKPPFGAISSSVLLMIIYTTFCDTFSNPNIDLDKFSL
Felis catus              157 FLGSSSSVPFTSIFSQLFMTVVVPLIIGQIVRRYIKDWLERKKPPFGAVSSCVLLTIIYTTFCDTFSNPNIDLDKFSL
Gallus gallus            157 FLGSSSSVPFTSIFSQLFMTVVVPLIIGQIVRRYIKDWLERRKPPFGTISSCVLLMIIYTTFCDTFANPNIDLDKFSL
Danio rerio              157 FLGSSSSVPFTSIFSQLFMTVVVPLIVGQVCRRFLRECLDRRKPPFGAVSSVVLLMIIYSTFCDTFNNPNIELDHLSL
Xenopus laevis           157 FLGSSSSVPFTSIFSQLFMTVVVPLIIGQIVRRYIKDWLERKKPPFGAISSCVLLMIIYTTFCDTFSNPNIDLDTFSL

Homo sapiens             235 VLILFIIFSIQLSFMLLTFIFSTRNNSGFTPADTVAIIFCSTHKSLTLGIPMLKIVFAGHEHLSLISVPLLIYHPAQI
Mus musculus             235 ILILFIIVSVQLSFMLLTFIFSTRNNSGFTPADTVAIIFCSTHKSLTLGIPMLKIVFAGHEHLSLISVPLLIYHPAQI
Rattus norvegicus        235 ILILFIIVSIQLSFMLLTFVFSTRNNSGFTPADTVAIIFCSTHKSLTLGIPMLKIVFAGHEHLSLISLPLLIYHPAQI
Bos taurus               235 LLVLFIICSIQLSFMLLTFIYSTRNNSGFTPADTVAVMFCSTHKSLTLGIPMLKIVFAGHEYLSLISVPLLIYHPVQI
Sus scrofa               235 ILILFTICSIQLSFMLLTFIFSTRNNSGFTPADTVAIMFCSTHKSLTLGIPMLKIVFAGHEHLSLISVPLLIYHPVQI
Equus caballus           235 ILILFIIFSIQLSFMLLTFIFSTRNNSGFTPADTVAIIFCSTHKSLTLGIPMLKIVFAGHEHLSLISVPLLIYHPVQI
Canis lupus familiaris   235 ILILFIIFSIQLSFMVLTFLFSTRNNSGFTPADTVAIIFCSTHKSLTLGIPMLKIVFAGHEHLSLISVPLLIYHPVQI
Felis catus              235 LLVLFIIFSIQLSFMLLTFLFSTRNNSGFTPADTVAIIFCSTHKSLTLGIPMLKIVFAGHEHLSLISVPLLIYHPVQI
Gallus gallus            235 IIIVFIIFSVQMSFMFLTFLFSTRSNSGFTPADTVAIIFCSTHKSLTLGIPMLKIVFAGYEHLSLISVPLLIYHPAQI
Danio rerio              235 LTVVFIIFSIQLSFMALIFFLSTRKSSGFSAADSVAIMFCATHKSLTLGIPMLKIVFEGYEHLSLISVPLLIYHPAQI
Xenopus laevis           235 VVIVFIIFFIQLAFMLLTFLFSTSKNSGFTPADTVAIVFCSTHKSLTLGIPMLKIVFVGYEHLSLISVPLLIYHPAQI

Homo sapiens             313 LLGSVLVPTIKSWMVSRQKGVKLTRPTV-----
Mus musculus             313 LLGSVLVPTIKSWMVSRQKGVKLTRPTV-----
Rattus norvegicus        313 LLGSVLVPTIKSWMVSRQKGVKLTRPTV-----
Bos taurus               313 LLGSVLVPTIKSWMVSRQKGVKLMRPTV-----
Sus scrofa               313 LLGSVLVPTIKSWMVSRQKGVKLTRPTV-----
Equus caballus           313 LLGSVLVPTIKSWMVSRQKGVKLTRPTV-----
Canis lupus familiaris   313 LLGSVLVPTIKSWMVSRQKGVKLTRPTV-----
Felis catus              313 LLGSVLVPTIKSWMVSRQKGVKLTRPTV-----
Gallus gallus            313 LLGSLLVPTIKSWMVSRQKALKLTRQPKVPVKV
Danio rerio              313 LLGSVLLPSIKTWMSGRQKTLTPI---------
Xenopus laevis           313 LLGSVLVPTIKSWMLSRQKALKLTRQPKIPL--
```

SLC10A7 transcript variant 2

(f)

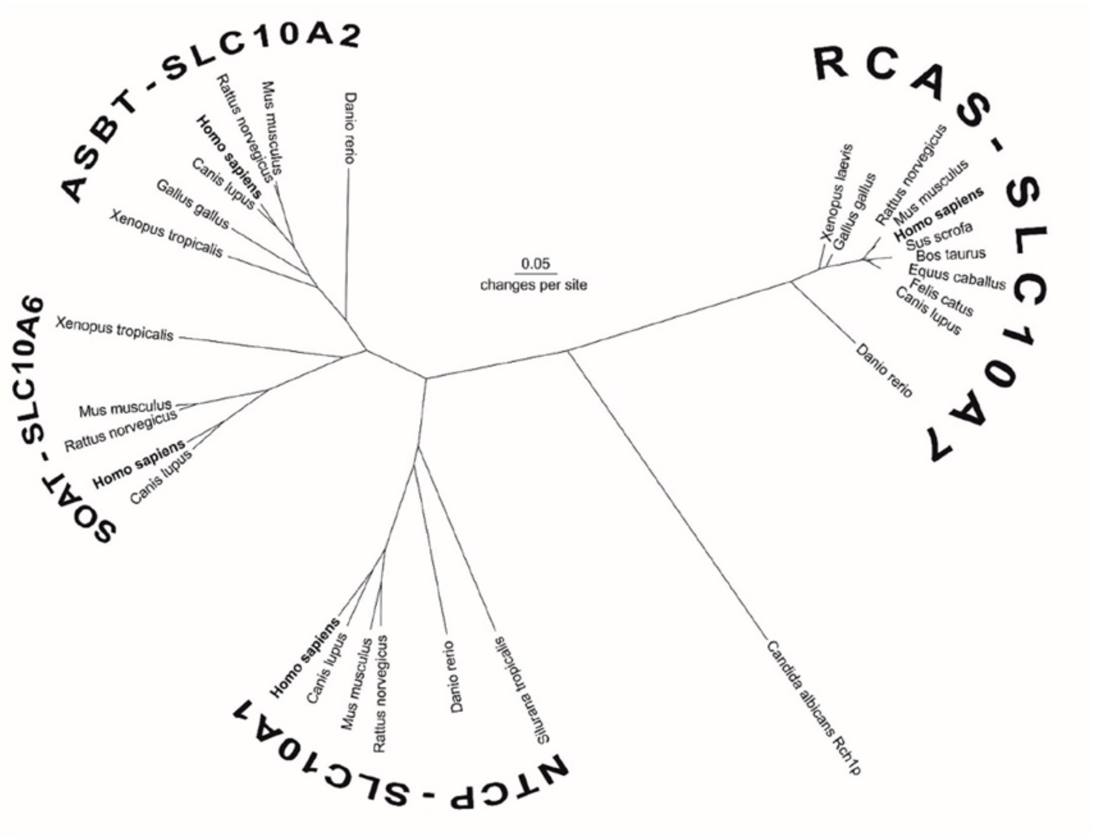

(g)

% identity	Homo sapiens	Mus musculus	Rattus norvegicus	Bos taurus	Sus scrofa	Equus caballus	Canis lupus familiaris	Felis catus	Gallus gallus	Danio rerio	Xenopus laevis
Homo sapiens	100.0	94.6	94.3	94.0	95.5	98.2	97.0	96.7	88.7	75.6	87.8
Mus musculus	94.6	100.0	97.6	91.7	93.8	95.2	93.5	93.5	87.5	75.0	86.0
Rattus norvegicus	94.3	97.6	100.0	90.8	92.9	94.3	93.5	93.5	86.9	75.3	86.3
Bos taurus	94.0	91.7	90.8	100.0	95.8	94.9	93.8	95.5	84.8	75.3	84.2
Sus scrofa	95.5	93.8	92.9	95.8	100.0	96.7	95.5	96.1	87.5	76.2	86.0
Equus caballus	98.2	95.2	94.3	94.9	96.7	100.0	98.2	97.0	89.6	76.2	88.1
Canis lupus familiaris	97.0	93.5	93.5	93.8	95.5	98.2	100.0	97.0	89.0	75.9	86.9
Felis catus	96.7	93.5	93.5	95.5	96.1	97.0	97.0	100.0	88.1	76.8	87.2
Gallus gallus	88.7	87.5	86.9	84.8	87.5	89.6	89.0	88.1	100.0	78.9	92.0
Danio rerio	75.6	75.0	75.3	75.3	76.2	76.2	75.9	76.8	78.9	100.0	80.1
Xenopus laevis	87.8	86.0	86.3	84.2	86.0	88.1	86.9	87.2	92.0	80.1	100.0

Supplementary Fig. S2. SLC10A7 transcript variant v2. (**a**) Exon composition of SLC10A7 transcript variant v2. The open reading frame consists of 1023 bp and is composed of 12 coding exons. The coded protein (isoform b) consists of 340 amino acids and is regarded as the full-length SLC10A7 reference protein. (**b**) Exon-intron organization of the human *SLC10A7* gene coding for SLC10A7 transcript variant v2. Lengths of all exons and introns are provided in addition to the nucleotide sequences at the exon/intron boundaries. Exon sequences are shown in uppercase, intron sequences in lowercase letters. (**c**) Schematic membrane topology model of the human SLC10A7 protein isoform b with ten transmembrane domains (TMDs) encoded by the 12 exons. The N- and C-terminal ends are both located intracellularly. Numbers indicate the amino acid positions at the beginning and end of each TMD. (**d**) Side and top view of a 3D homology model of human SLC10A7 isoform b. The SLC10A7 protein sequence with GenBank accession Number NP_001025169 was used as a target sequence for the SWISS-MODEL tool [4]. The model was calculated based on the template of ASBT from Yersinia frederiksenii (PDB: 4n7w) [5]. (**e**) Alignment of homologous SLC10A7 proteins from different species. The sequences correspond to the following GenBank Accession Numbers: NP_001025169.1 (human), NP_084012.1 (mouse), NP_001010948.1 (rat), XP_010812021.2 (cattle), NP_001131112.1 (swine), XP_001501868.1 (horse), XP_855172.2 (dog), XP_003985015.1 (cat), XP_015131664.1 (chicken), NP_001003420.1 (zebrafish), and NP_001080729.1 (African clawed frog). (**f**) Sequence distance and phylogenetic relationship of selected members of the SLC10 carrier family from different species. (**g**) Distance table of homologous SLC10A7 proteins from different species, showing the percent identity.

SLC10A7 transcript variant 4

(a)

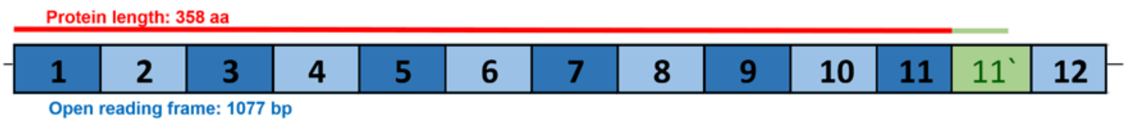

(b)

Exon	CDS (bp)	5`-Splice donor	3`-Splice acceptor	Intron size (kb)
1	100	ATGGGG/gtaagt	ttttag/GACCAC	4.4
2	83	ACAGAG/gtactg	ttccag/GAGCTG	6,9
3	137	AAAAGG/gtatgt	tttcag/TTTGCA	5.9
4	76	AATGAG/gtgagt	tcatag/GCAGCT	61.0
5	39	TTTTTG/gtaagt	tttcag/GGCATC	116.7
6	36	CTTTTT/gtgagt	ttacag/CTTGGT	19.9
7	84	GGACAG/gtaagg	tttcag/ATTGTC	11.8
8	166	TCATAA/gtaagt	cttcag/TATTTT	0.9
9	52	AACAAG/gtaagt	ccctag/GAATAA	9.6
10	74	CATTGG/gtaagt	caacag/GAATTC	24.3
11	146	CAGAAG/gtgagt	gtgtag/AAACTA	1.7
11`	84	CATTAA		

(c)

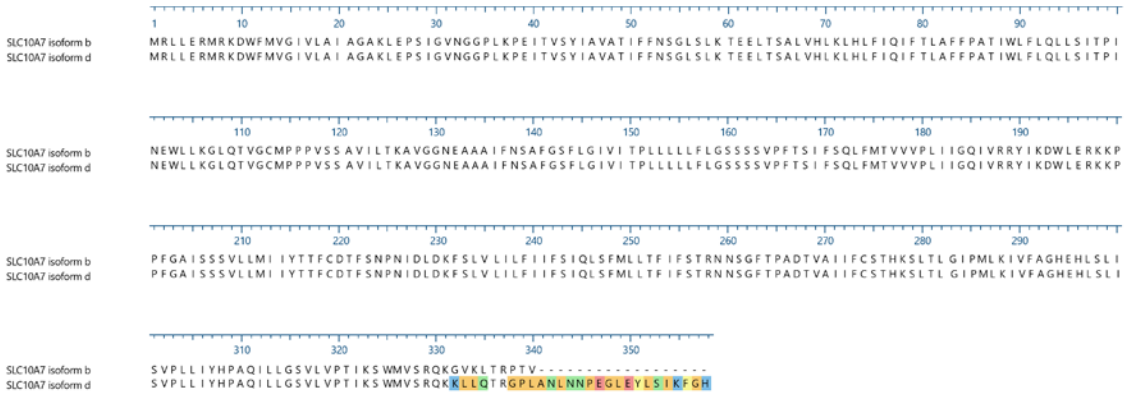

(d)

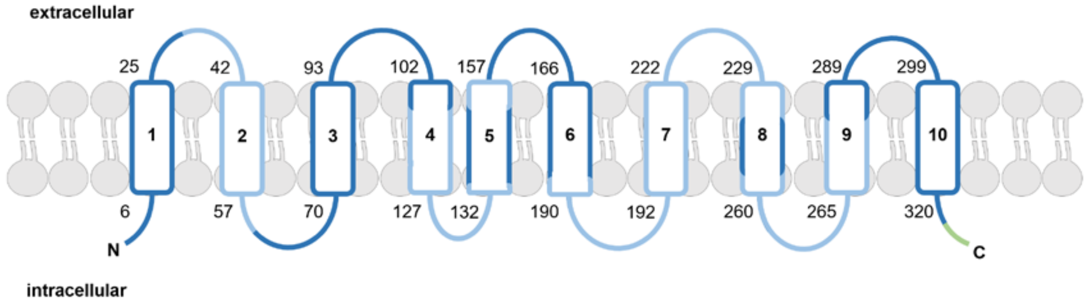

Supplementary Fig. S3. SLC10A7 transcript variant v4. (**a**) Exon composition of SLC10A7 transcript variant v4. The open reading frame consists of 1077 bp and is composed of 12 coding exons. The coded protein (isoform d) consists of 358 amino acids and differs from isoform b only in the C-terminal sequence that is coded by an alternative exon 11'. Exon 12 is non-coding in SLC10A7 transcript variant v4. (**b**) Exon-intron organization of the human *SLC10A7* gene coding for SLC10A7 transcript variant v4. Lengths of all exons and introns are provided in addition to the nucleotide sequences at the exon/intron boundaries. Exon sequences are shown in uppercase, intron sequences in lowercase letters. (**c**) Protein alignment of SLC10A7 isoform b and d. Both proteins only differ in their C-terminal amino acids. (**d**) Schematic membrane topology model of human SLC10A7 isoform d with ten TMDs encoded by exons 1-11 and 11'. The N- and C-terminal ends are both located intracellularly. Numbers indicate the amino acid positions at the beginning and end of each TMD.

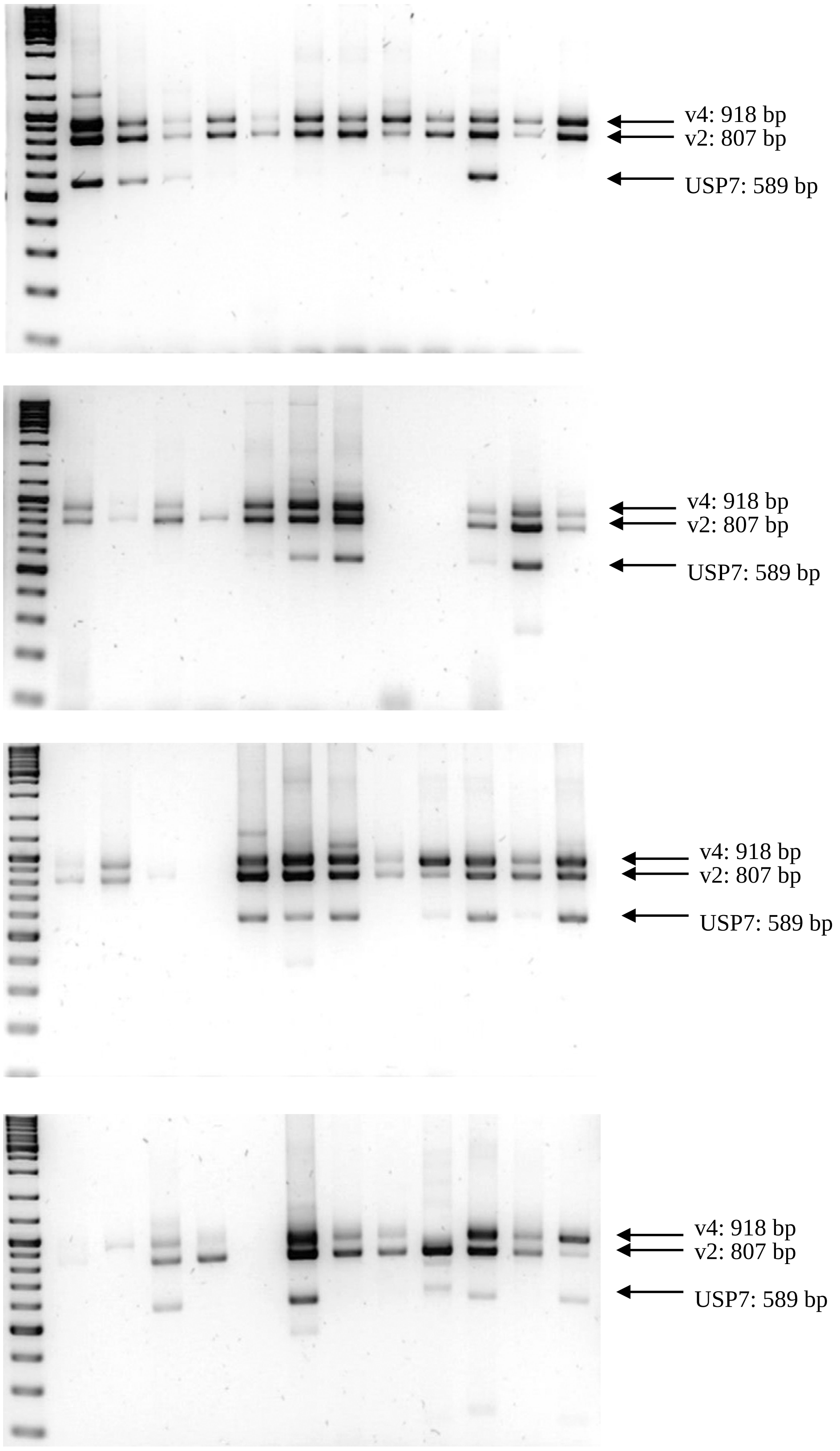

Supplementary Fig. S4. Full-length gels corresponding to Fig. 6. Expression pattern of SLC10A7 transcript variants v2 and v4 in different human tissues. Expression analysis was performed on a commercial major human tissue cDNA panel with primers theoretically allowing amplification of different SLC10A7 transcript variants. Primers were designed to bind at exon boundary 3/4 (forward) and at exon 12 (reverse). Amplicons were separated on a 2.5% agarose gel. Individual bands were excised from the gel and subjected to DNA sequencing. Only SLC10A7 transcript variants v2 (amplicon size: 807 bp) and v4 (amplicon size: 918 bp) were detected. The smaller band at 589 bp revealed to be unspecific and corresponds to homo sapiens ubiquitin specific peptidase 7 (USP7), transcript variant 3 (GenBank Accession Number NM_001286458).

References

1. Ashikov, A. *et al.* Integrating glycomics and genomics uncovers SLC10A7 as essential factor for bone mineralization by regulating post-Golgi protein transport and glycosylation. *Hum Mol Genet* **27**, 3029-3045 (2018).

2. Dubail, J. *et al.* SLC10A7 mutations cause a skeletal dysplasia with amelogenesis imperfecta mediated by GAG biosynthesis defects. *Nat Commun* **9**, 3087 (2018).

3. Laugel-Haushalter, V. *et al.* A New SLC10A7 Homozygous Missense Mutation Responsible for a Milder Phenotype of Skeletal Dysplasia With Amelogenesis Imperfecta. *Front Genet* **10**, 504 (2019).

4. Arnold, K., Bordoli, L., Kopp, J., & Schwede, T. The SWISS-MODEL workspace: a web-based environment for protein structure homology modelling. *Bioinformatics* **22(2)**, 195-201 (2006).

5. Zhou, X. et al. Structural basis of the alternating-access mechanism in a bile acid transporter. *Nature* **505(7484)**, 569-573 (2014).
